# Image-based Evaluation of Cutting Forces During Ultrasonic Cutting of Bone

**DOI:** 10.1007/s11340-025-01256-0

**Published:** 2025-12-09

**Authors:** A. Marek, X. Li, M. Lucas, F. Pierron

**Affiliations:** 1 Faculty of Engineering and Physical Sciences, University of Southampton, University Road, Southampton, SO17 1BJ United Kingdom; 2 Centre for Medical and Industrial Ultrasonics, James Watt School of Engineering, University of Glasgow, Glasgow, G12 8QQ United Kingdom; 3 MatchID NV, Leiekaai 25A, 9000 Ghent, Belgium

**Keywords:** Virtual fields method, Digital image correlation, Ultrasonic cutting, Force identification

## Abstract

**Background:**

Ultrasonic surgical cutting tools are an appealing alternative to traditional devices. The measurement of time-resolved ultrasonic cutting forces is important to understand the material removal process but a challenge because of inertial effects affecting the results of traditional load cells.

**Objective:**

The present work details an attempt to resolve the cutting force of an ultrasonically powered surgical tool at the time scale of the 25 kHz ultrasonic excitation, by using the tool tip itself as the load cell.

**Methods:**

Ultra-high speed imaging is used in conjunction with Digital Image Correlation to measure strain and acceleration in the cutting blade. Dynamic equilibrium is then leveraged to identify the cutting force at the tool tip within a single cutting cycle of the tool. The methodology is first verified on simulated data before being applied to experiments on cortical bovine bone (free vibrations and cutting).

**Results:**

The force detection threshold was evaluated on the order of 5 N, recorded at 1 Mfps with a Shimadzu HPV-X camera. A particular difficulty was that the blade exhibited some out-of-plane bending which created a bias on the identified cutting force. Finally, it was possible to detect that when the blade engaged in the workpiece, a compressive force acted on the tool while during the disengagement part of the cycle, the force was null.

**Conclusions:**

It was shown that it was possible to use the thin flat blade of an in-house developed ultrasonic surgical tool to measure time-resolved cutting forces.

## Introduction

Bone incisions and resections in surgical procedures require high precision, minimal micro-damage of the bone beyond the cut site and minimal damage to surrounding soft tissue structures. Conventional methods of bone cutting rely on powered tools, which induce large cutting forces and high temperatures that lead to microcracks, thermal necrosis and trauma [1–4]. These are detrimental to patient outcomes and can delay the healing process. Ultrasonic bone scalpels employ high-frequency vibrations and are tuned to a low ultrasonic frequency, between 20 and 35 kHz. These surgical instruments cut bone tissue with high precision and low force. They are tissue selective and hence do not damage surrounding soft tissue structures [5, 6]. Despite these advantages and the growing clinical need for ultrasonic surgical devices, the dynamic interaction between the surgical tip and bone tissue lacks fundamental understanding. Measurement of the dynamic cutting force during interaction of an ultrasonic cutting blade and bone is challenging, yet is important for the design of effective surgical devices.

Traditional cutting force measurement methods adopt direct sensor-based techniques, such as dynamometers or load-cells. Although these provide high accuracy, they often interfere with the cutting dynamics and are challenging to integrate into a surgical environment [7, 8]. Moreover, located further away from the tool tip, they will generally record inaccurate force in situations where transient dynamics are present, which is the case in ultrasonic cutting. As a consequence, they can only measure the low frequency force component but not the time-resolved component at the ultrasonic excitation frequency. There is therefore a need for a non-contact method of cutting-force measurement that can resolve the force in a cycle of ultrasonic vibration, denoted here ‘time-resolved’.

Deformation imaging techniques like Digital Image Correlation (DIC, [9]) offer new opportunities to analyse cutting processes. For instance, Baizeau et al. [10] used a double exposure sCMOS camera together with a pulsed laser to obtain strain maps during orthogonal cutting and residual strain maps after cutting. In a related paper [11], they used an analytical solution to the displacement at the tip of the tool to obtain the steady-state cutting force. A similar study was performed by Huang et al. [12] using a camera with 2000 fps, calculating the cutting force from the strain maps and comparing it with load cell measurements and simulation. These studies concentrate on steady-state orthogonal cutting and do not consider transient wave effects; as such, they are not directly relevant to the present work.

The recent development of ultra-high speed imaging has opened up opportunities to measure deformations in transient dynamics situation, where inertial effects are present. In fact, for material identification, acceleration can be leveraged to avoid the need for external force measurement [13]. The concept has been recently applied to the identification of transient surface pressures acting on a plate, caused either by air pressure (blower) [14], blast [15] or wave slamming [16]. In this corpus of work, the Virtual Fields Method (VFM) [17] was used to identify the load distribution from strain and acceleration maps.

In the field of material response to ultrasonic loading, seminal work was published by Wang et al. [18] where DIC was successfully applied to measure the full-field displacement response of bone mimic material samples subject to ultrasonic excitation at frequencies typical of surgical devices. The technique captured the oscillatory displacement cycles, demonstrating its effectiveness for characterising power ultrasonic applications. This was later adapted to obtain the mechanical behaviour of materials [19, 20], including cortical bovine bone [21]. However, in these studies, measurements only captured the response of the sample mounted on an ultrasonic transducer and were therefore not representative of ultrasonic cutting.

DIC has since been adopted in a study of ultrasonically assisted cutting of bone, where the cutting set-up is based on a material removal configuration akin to metal machining. Strain and strain rate measured using DIC was used to calculate cutting force [22–24] and ultimately to validate a cutting force model. However, these studies did not resolve the timescales of the ultrasonic excitations

The present work details an attempt to resolve the cutting force of an ultrasonically powered surgical tool at the time scale of the 25 kHz ultrasonic excitation. It leverages the availability of ultra-high speed imaging and deformation measurements through Digital Image Correlation. Finally, dynamic equilibrium of the cutting blade allows to derive the cutting force. The first section details the identification method, the second explains the methodology which is articulated around a first verification and validation step using simulated data, and secondly, an experimental campaign on bovine cortical bone. Finally, results are provided and discussed.

## Theory

### Cutting Force from Kinematic Fields

**Fig. 1:**
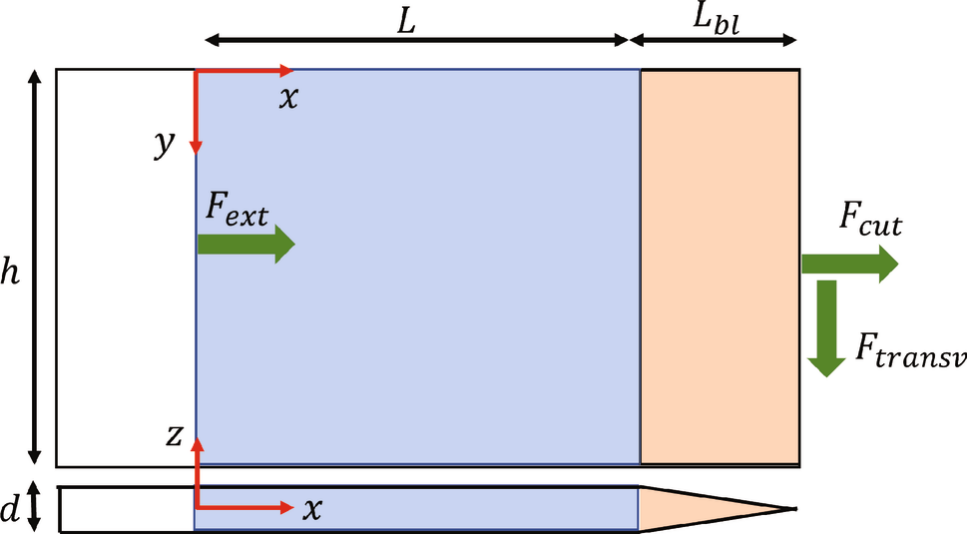
A schematic of blade geometry with annotated acting forces

At any point in time, all points in a body satisfy the dynamic stress equilibrium, which can be expressed in the strong form as:

1 $$\begin{aligned} \mathbf {\nabla } \cdot \boldsymbol{\sigma } + \boldsymbol{b} = \rho \boldsymbol{a} \end{aligned}$$

where $\boldsymbol{\sigma }$ is the stress tensor, $\boldsymbol{b}$ is the body forces vector, $\boldsymbol{a}$ is the vector of accelerations and $\rho$ is the mass density. Equivalently this principle can be expressed in a weak form (called the principle of virtual work, PVW) which is expressed as follows when body forces are neglected:

2 $$\begin{aligned} \overbrace{\int _{\partial V} \boldsymbol{f} \cdot \boldsymbol{u}^{*} \mathop {d\partial V}}^{\text {EVW}} - \overbrace{\int _{V} \boldsymbol{\sigma }:\boldsymbol{\varepsilon }^{*} \mathop {dV}}^{\text {IVW}} = \overbrace{\int _{V} \rho \boldsymbol{a} \cdot {u}^{*} \mathop {dV}}^{\text {AVW}} \end{aligned}$$

where $\boldsymbol{f}$ is the traction vector at the boundary of the solid $\partial V$, *V* is the volume of the body and starred values: $\boldsymbol{u}^{*}$, $\boldsymbol{\varepsilon }^{*} = \nabla \boldsymbol{u}^{*}$ are test functions called virtual displacements and virtual strains respectively. These test fields can be any functions, as long as the virtual displacement is continuous and piecewise differentiable. The principle of virtual work consists of three terms, called virtual work of external forces (EVW), internal forces (IVW) and accelerations (AVW). The virtual fields method (VFM) is an inverse identification technique [17] that leverages the PVW to obtain unknown material properties from known full-field kinematic maps that are obtained experimentally, but it can also be applied to a problem where material properties are known, but the traction forces are to be identified. Let us consider a scenario where a force acting at the tip of a cutting device is to be measured, as shown in Fig. 1, and an imaginary cut is done through the left edge of the blue area (portion where measurements are available). As any virtual displacement field can be selected in the PVW, one can define a virtual displacement such that the unknown section force $F_{ext}$ is cancelled from the equilibrium equation while the cutting force, $F_{cut}$ is not. One of such virtual displacement fields is shown below:

3 $$\begin{aligned} {\left\{ \begin{array}{ll} u_{x}^{*} = {\left\{ \begin{array}{ll} x,~\text {for } 0 \le x \le L \\ L,~\text {for }x > L \end{array}\right. } \\ u_{y}^{*} = 0 \end{array}\right. } \end{aligned}$$

In this case, the only non-zero virtual strain component is $\varepsilon _{xx}^{*} = 1$ for $0 \le x \le L$. Inputting such fields into equation (2) and evaluating each term separately yields:

4 $$\begin{aligned} \begin{aligned} \text {EVW}&= F_{ext}u_{x}^{*}(x=0) \\&+ F_{cut}u_{x}^{*}(x=L+L_{bl}) \\&= F_{cut}L \end{aligned} \end{aligned}$$

Here, we used the definition of section forces: $F = \int _{\partial V} \boldsymbol{f}\mathop {d\partial V}$ and the fact that the virtual displacement only depends on *x*. It should be noted that it is likely that the force distribution at the tip of the tool, and also over the lateral faces engaged in the tissue, will not be uniform but the use of this particular virtual field filters out this distribution to yield the resultant, regardless of that distribution. Likewise, any potential out-of-plane force component is also filtered out as there is no virtual out-of-plane displacement. The VFM has been used in the past to identify load distributions (for instance, [14]) but this would require larger deformations and better spatial resolution than in the present case.

To evaluate the volume integral of stresses, first an assumption must be made about the distribution of stresses through the thickness of the body. This is due to the fact that the available kinematic measurements are limited to the surface of the body. A typical assumption in such cases is of constant stress through the thickness, with a plane stress assumption. The stresses are inferred from strains measured with digital image correlation, which produces a dense grid of points, therefore the integral can be approximated with a discrete sum:

5 $$\begin{aligned} \text {IVW} = \int _{V} \boldsymbol{\sigma }:\boldsymbol{\varepsilon }^{*} \mathop {dV} \approx d \sum _{i=1}^{n_{pts}} \sigma _{xx}^{(i)}.{1}.{A^{(i)}} \end{aligned}$$

where *d* is the thickness and $A^{(i)}$ is the surface area corresponding to the *i*-th strain measurement point. The equation can be further simplified by defining the average value $\bar{(.)} = \frac{1}{n} \sum _{i}^{n} (.)_{i}$ across the whole region of interest and invoking Hooke’s law for plane stress, assuming that the blade behaves as an isotropic homogeneous linear elastic body:

6 $$\begin{aligned} \text {IVW} = dLh\overline{\sigma _{xx}} = dLh(Q_{xx}\overline{\varepsilon _{xx}^{memb}}+Q_{xy}\overline{\varepsilon _{yy}^{memb}}) \end{aligned}$$

where $\boldsymbol{\varepsilon }^{memb}$ is the membrane strain tensor and $Q_{xx} = \frac{E}{1-\nu ^2}$, $Q_{xy} = \nu Q_{xx}$ are plane stress stiffnesses that depend on Young’s modulus (*E*) and Poisson’s ratio ($\nu$). Typically, it is assumed that the membrane strain is the same as the measured surface strain, however this is not necessarily true if bending effects are present, *e.g.* due to parasitic modes being excited. In thin plate theory, the strain tensor can be written as:

7 $$\begin{aligned} \boldsymbol{\varepsilon }(z) = \boldsymbol{\varepsilon }^{\text {memb}} - z \boldsymbol{\kappa } \end{aligned}$$

where $\boldsymbol{\kappa } = \boldsymbol{H}(w)$ is the curvature tensor (Hessian of *w*) that can be determined experimentally when using stereo DIC; *w* and *z* are the out-of-plane displacement and coordinate respectively. Equation (7) can be written for $z=\dfrac{-d}{2}$ so that the membrane strain is estimated based on the available surface measurements on the blue area in Fig. 1:

8 $$\begin{aligned} \boldsymbol{\varepsilon }^{\text {memb}} = \boldsymbol{\varepsilon }^{\text {surf}} + \frac{-d}{2} \boldsymbol{\kappa } \end{aligned}$$

Finally, the AVW integral can be evaluated by splitting the body into two parts: the region of interest where kinematics are measured and the overhang volume where the kinematics are unknown. Again, an assumption about constant through-thickness acceleration has to be made to evaluate the volume integral:

9 $$\begin{aligned} \begin{aligned} \text {AVW}&= \rho \int _{V} \boldsymbol{a}\cdot \boldsymbol{u}^{*} \mathop {dV} \\&=\rho dh\int _{0}^{L} a_{x}x \mathop {dx} \\&+ \rho h\int _{-d/2}^{d/2}\int _{L}^{L+L_{bl}} a_{x}L \mathop {dx}\mathop {dz} \\&\approx \rho dLh\overline{a_{x}x} + \rho L V_{overhang} \overline{a_{x}} \end{aligned} \end{aligned}$$

Since the accelerations are not measured directly on the overhang portion of the body, they are approximated as the mean acceleration over the region of interest, which is motivated by small spatial variation in the acceleration field in the considered case (vibration rather than wave propagation).

Adding the three virtual work terms together, as presented in equation (2) and defining the volume of the blue region as $V = dLh$, yields:

10 $$\begin{aligned} F_{cut}L - V(Q_{xx}\overline{\varepsilon _{xx}} +Q_{xy}\overline{\varepsilon _{yy}}) =\rho V \overline{a_{x}x} + \rho L V_{overhang} \overline{a_{x}} \end{aligned}$$

A simple algebraic manipulation leads to the final equation for calculating the cutting force:

11 $$\begin{aligned} F_{cut} =\frac{V}{L}\left( Q_{xx}\overline{\varepsilon _{xx}}+Q_{xy}\overline{\varepsilon _{yy}}\right. +\left. \rho V \overline{a_{x}x}\right) + \rho V_{overhang} \overline{a_{x}} \end{aligned}$$

The cutting force $F_{cut}$ can be directly reconstructed from surface measurements of displacements over the region of interest, given that the material properties (*E*, $\nu$ and $\rho$) of the blade are known, and spatial averages based on strains and acceleration over the blue surface of Fig. 1 can be evaluated from full-field deformation measurements with Digital Image Correlation (DIC). During the tool vibration, images of the blade are taken using high-speed imaging. Then, DIC is used to calculate full-field displacements from the images, which are subsequently differentiated spatially, to get surfaces strains, then membrane strains with equation (8), and temporally, to obtain accelerations. These data are then inserted into equation (11) and the cutting force, $F_{cut}(t)$ is reconstructed.

### Ultrasonic Cutting Device

Power ultrasonic transducers can be represented mathematically using a simplified mechanical model consisting of a mass, a dashpot and a spring element. The governing equation of this system can be expressed as a second order differential equation as shown below:

12 $$\begin{aligned} m \frac{d^2 u}{\mathop {dt}^2} + c \frac{du}{\mathop {dt}} + ku = F_{0}\exp (i\omega t) \end{aligned}$$

where *u* is the displacement, *m* is the mass constant, *c* is the dashpot constant, *k* is the spring constant and $F_{0}\exp (i\omega t)$ is the driving force at frequency $\omega$. Such system has some resonance frequency for which the response is maximized when the driving force frequency matches it.

The transducer is driven with alternating current supplied to the electrodes sandwiching the piezoelements. A simplified model of this electrical circuit consists of an inductor, a resistor and a capacitor connected in series. In this case, the governing equation of the system is a second order differential equation shown below:

13 $$\begin{aligned} L \frac{\mathop {dI}}{\mathop {dt}} + RI + C\int {I\mathop {dt}} = V_{0}\exp (i\omega t) \end{aligned}$$

where *L* is the inductance, *I* is the current, *R* is the resistance, *C* is the capacitance and $V_0$ is the voltage.

Ultrasonic transducers are electro-mechanical devices where the two systems interact with each other. Often, this system is modelled using the Butterworth Van-Dyke model linking the two parts. By comparing equations (12) and (13), one can relate how the displacement (mechanical part) interacts with the current (electrical part):

14 $$\begin{aligned} F \propto V \end{aligned}$$

15 $$\begin{aligned} \frac{dx}{dt} \propto I \end{aligned}$$

The driving force is proportional to the applied voltage and the tool velocity is proportional to the current.

**Fig. 2:**
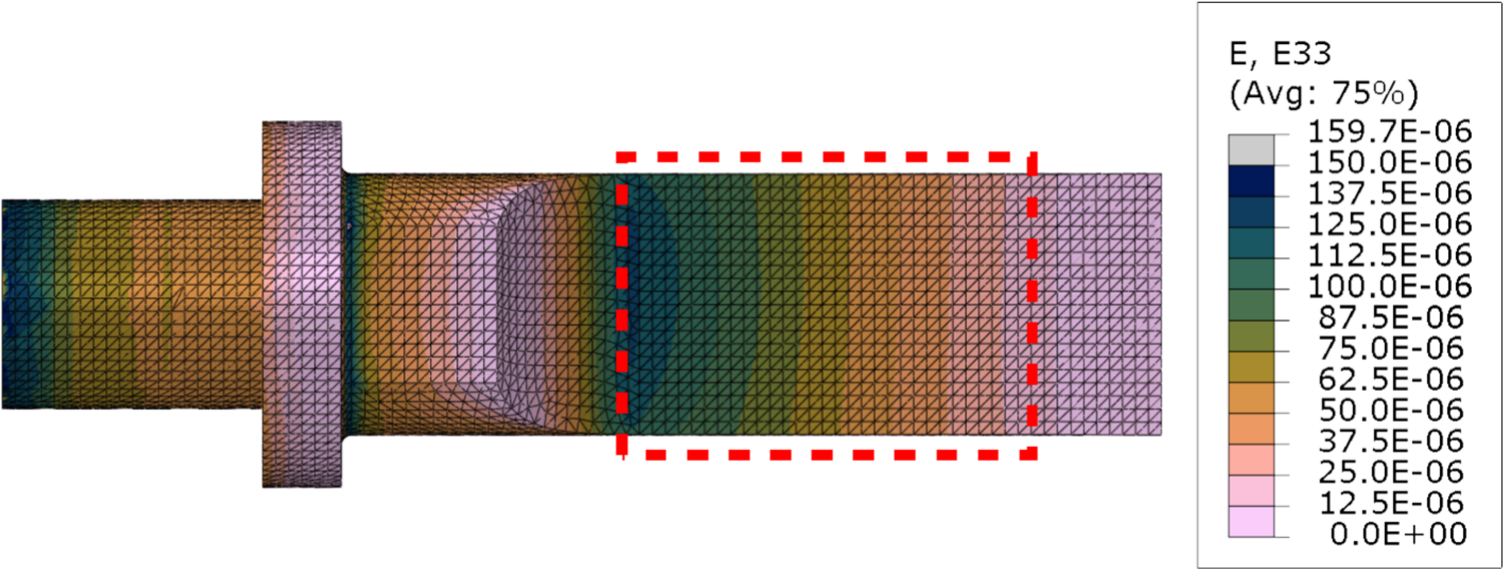
Longitudinal strain amplitudes across the blade part of the finite element model (22.183 kHz), when the vibration amplitude is 12.5 µm. The dashed area represents the flat area of the blade over which displacement measurements are taken

## Methodology

### Modal Analysis

To test the proposed method of force measurement, first a finite element model of the cutting device was prepared in Abaqus (SIMULIA). The model provided synthetic data (displacements) comparable to those acquired during a real experiment. The verification process was split into two parts: in the first part, no cutting force was present and the displacement fields represented only the steady-state (free) vibration of the blade. In the second stage, a dynamic force was superimposed onto the model to represent the cutting force acting on the tip of the blade, as it extends into the material.

The model was defined using the 3D geometry based on CAD drawings of the in-house built ultrasonic tool, operating at approximately 25 kHz, that was used in this study. All major parts of the assembly (such as piezoceramic discs, back and front masses, fasteners) were modelled using 3D, 10-node quadratic tetrahedrons (C3D10) and were assigned relevant material properties (elasticity, mass density and damping).

#### Free vibration conditions

The displacement fields corresponding to the design frequency were calculated by first evaluating the natural frequencies of the tool between 20 and 30 kHz. The first longitudinal resonance mode was identified at 22.183 kHz. A steady-state analysis at this frequency was then run, with a single force (10 N) applied to the centre of the tip of the blade, in the longitudinal direction of the tool. The complex displacement amplitudes ($\boldsymbol{u}^{SSD}$) were extracted from the flat part of the blade (see Fig. 2) and used to construct kinematic fields corresponding to a fictitious camera observing the experiment according to the following formula:

16 $$\begin{aligned} \boldsymbol{u} = |\boldsymbol{u}^{SSD} |\cos \left( 2\pi f \frac{n_{frame}}{f_{image}} + \arctan \left( \frac{{\text {Im}}(\boldsymbol{u}^{SSD})}{{\text {Re}}(\boldsymbol{u}^{SSD})} \right) \right) \end{aligned}$$

where $n_{frame}$ is the number of the generated image and $f_{image}$ is the camera acquisition frequency.

#### Added cutting force

To simulate cutting force, a simplified approach was taken using the superposition rule. Using the same finite element model as before, the response of the blade to a dynamic force acting on the tip was investigated. The force was applied to a reference point that was kinematically tied to the entire cutting edge. The value of the force varied with time according to a rectified half sine wave, *i.e.* during the positive half of period the force followed a standard sine wave, while in the negative half it was null. Such approach was adopted to simulate the scenario in which the blade contacts intermittently the material and applies force, followed by retraction of the blade and loss of contact during which no force is applied. The model was evaluated using implicit dynamics solver and the resulting displacement fields were superimposed with those from free vibration to produce complex displacement response encoding vibration and impact.

### Data Processing Pipeline

#### Digital image correlation

Digital image correlation is a well established method for measuring motion and shape of objects from greyscale images [9]. It relies on the principles of conservation of the optical flow and yields sub-pixel accuracy of typically 0.01 px. The displacements are calculated by splitting the image into a large number of small facets (called subsets), of typical linear sizes 15 px to 25 px and tracking their motion between pairs of images. The subsets can overlap each other, and the spacing (in number of pixels) between the centres of two consecutive subsets is called the step size. To make sure that each subset can be uniquely detected, the specimen is decorated with a random pattern which can be achieved in a number of different ways, such as using spray paint, an airbrush, printers or transferring techniques such as stamping.

Digital image correlation implementations typically rely on a number of internal parameters that affect metrological outcomes such as spatial resolution or noise floor of a variable. It is beyond the scope of this contribution to review the details of the method and the interested reader can refer to *A good practices guide for digital image correlation* [25]. The combination of the selected parameters strongly affects the accuracy of the measurements, therefore the measured force. In the proposed method, the force measurement relies mostly on two quantities measured experimentally: strains and accelerations, both of which are derived from the displacement fields by differentiating them in space or time. Among the parameters affecting the quality of reconstructed displacement fields are: subset size and subset shape function. The former leads to smaller noise floor when increased, but at the cost of reduced spatial resolution. The latter is used to map the reference subset shape (square) onto the deformed image, which is necessary to perform accurate matching. The shape function is typically a polynomial, with higher spatial resolution and noise floor, as the order of the polynomial increases.

In practice, recorded images are loaded into a commercial DIC package, MatchID (v.2023.1.0, MatchID NV), and correlated to produce displacement maps that can then be extracted to Matlab for further processing.

**Fig. 3:**
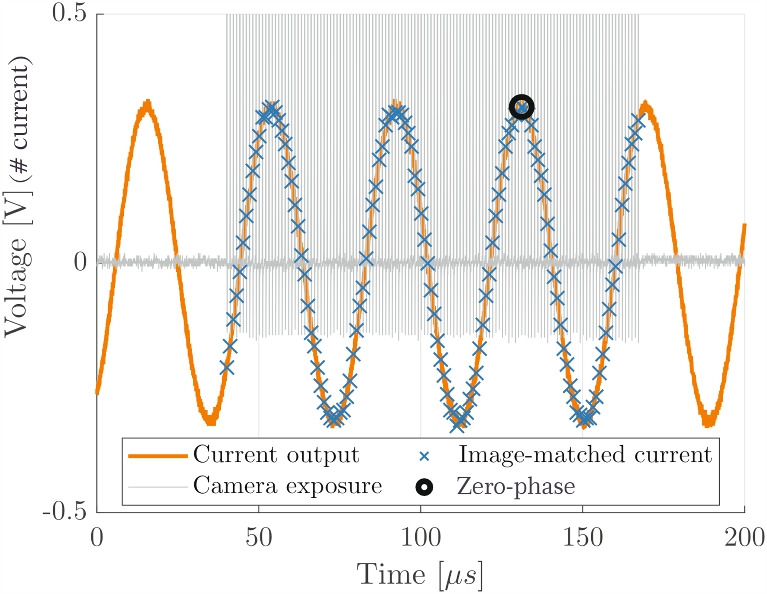
Camera synch, vertical axis voltage reflects current measurement

#### Estimation of the static configuration

Digital image correlation produces relative displacements with respect to the reference image. Typically, such image is taken when a body is in the unloaded state, so that the output of DIC is the absolute deformation. Such an approach is not viable when 2D DIC is used in this study, as even minute motion between when the reference is taken before the start of the ultrasonic excitation and when the experiment runs would cause spurious deformation (*e.g.* due to the out-of-plane motion) that could spoil the analysis. Instead, the collected images are correlated against one of the images that is closest to the zero-phase configuration. There are multiple ways this image can be selected, *e.g.* by correlating all the images against a random one, and finding the reference image as the one whose average longitudinal displacement is the closest to the mean displacement across all the images.

A more rigorous approach, that was adopted in this work, relies on using the proportionality between the velocity of the blade and the driving current (equation (15)). During the experiment the driving current and the exposure of the camera are monitored with an external device. During recording, the camera sends short 200 ns pulses whenever an image is taken, as shown in Fig. 3, in which the vertical axis shows the voltage output of the current monitoring device, so is proportional to the device current. These pulses can be then linked to the recorded driving signal and a current value can be assigned to each image. The set of current values is then cosine-fitted in time to assign phase of cycle to each image. When the current value has a phase of $\pi /2$ [$\pi$], then the displacement has a phase of 0 [$\pi$] therefore the zero-phase displacement configuration is found by identifying the image corresponding to peak current by looking for the phase value closest to $\pi /2$ [$\pi$].

#### Time registration of stereo images

When using the stereo camera configuration, the data could not be sufficiently time resolved to calculate continuous accelerations, due to the insufficient frame rate of the available cameras. To overcome this problem, a synthetic signal was reconstructed from the measurements according to the following procedure. The images were taken at a frame rate of 75 kfps, much smaller than the one required for temporally-resolving the displacements. Each image was matched to its corresponding current level, as outlined in the previous section and a cosine function was fitted to the current vs time data using the least-square method. Then, for each image, the phase of the cycle was calculated as:

17 $$\begin{aligned} \Phi (t) \equiv (\omega t + \phi _{0}) [2\pi ] \end{aligned}$$

where *t* is time when the image was taken, $\omega$ is the identified circular frequency of the driving current and $\phi _{0}$ is the initial phase shift. Then, the images are sorted so that they are ordered according to the increasing phase. Finally, a fictitious time is generated, by dividing the sorted phases by the identified circular frequency. This process reorders the images as if they had been collected during only one cycle of vibration, with uneven sampling frequency, much higher than the camera frame rate, enabling calculation of accelerations.

#### Post-processing of DIC data

Once a set of images is correlated, it yields full-field maps of displacements, corresponding to each image. Strains and accelerations are then calculated by performing differentiation with respect to space and time respectively. The differentiation operation amplifies noise so it is very important to do it in such a manner that introduces the least amount of noise in the differentiated signal, without distorting it too much. A common strategy is to fit displacement fields locally with a polynomial and obtain derivatives through analytical formulas. In this scheme, the size of the fitting window and the order of the polynomial are selected to provide optimal compromise between spatial and measurement resolutions.

Strain maps were calculated independently for each image. Two methods of strain calculation were considered in this work: ’local‘ and ’global‘. The former is the typical approach, where for each subset, a window (strain window, SW) of the surrounding subsets is used to locally fit a 2D polynomial and calculate strains at the centre of it by spatial differentiation of its coefficients. The larger the strain window, the smaller the noise floor, at the cost of spatial resolution. A useful metric to compare strain windows across different subset (SS) and step (ST) sizes is so-called virtual strain gauge (VSG) [25], that is defined as:

18 $$\begin{aligned} VSG = \left( \left[ SW-1 \right] ST\right) + SS \end{aligned}$$

The VSG represents the size of the area (in pixels) that is used to calculate strain at a particular point. Some of the points close to the edges of the region of interest (ROI) will not have enough surrounding points to construct the full strain window. In that case, these points are excluded from the analysis, as unfilled strain window can lead to larger errors. In the ’global‘ approach, a single polynomial surface is fitted to all the data points in the region of interest. This leads to much stronger regularisation but provides data right to the edges.

Similarly, accelerations were calculated by fitting, for each pixel, the displacement evolution over time with a polynomial and taking the second derivative of the coefficients to obtain accelerations. In this case, the fitting window corresponds to the number of images selected for the filter. The order of the polynomial can also be selected, as long as it is larger than two. Just as in the case of the ’local‘ strain approach, frames close to the edges (the beginning and the end of the video) are excluded from the data analysis, as they exhibit much higher errors.

Finally, the curvature tensor can be determined by fitting a global 2^nd^ order polynomial to the out-of-plane displacement fields and calculating the second order derivatives.

#### Calculation of the cutting force

With strains and accelerations calculated from the displacement time-history, the cutting force can be obtained using equation (11). The only remaining unknown in the equation is the mean acceleration of the tapered end, which for simplicity was assumed to be equal to the mean value of acceleration over the flat part, as there is very little spatial variation in the displacement/acceleration fields.

### Selecting of Optimal DIC Parameters

**Fig. 4:**
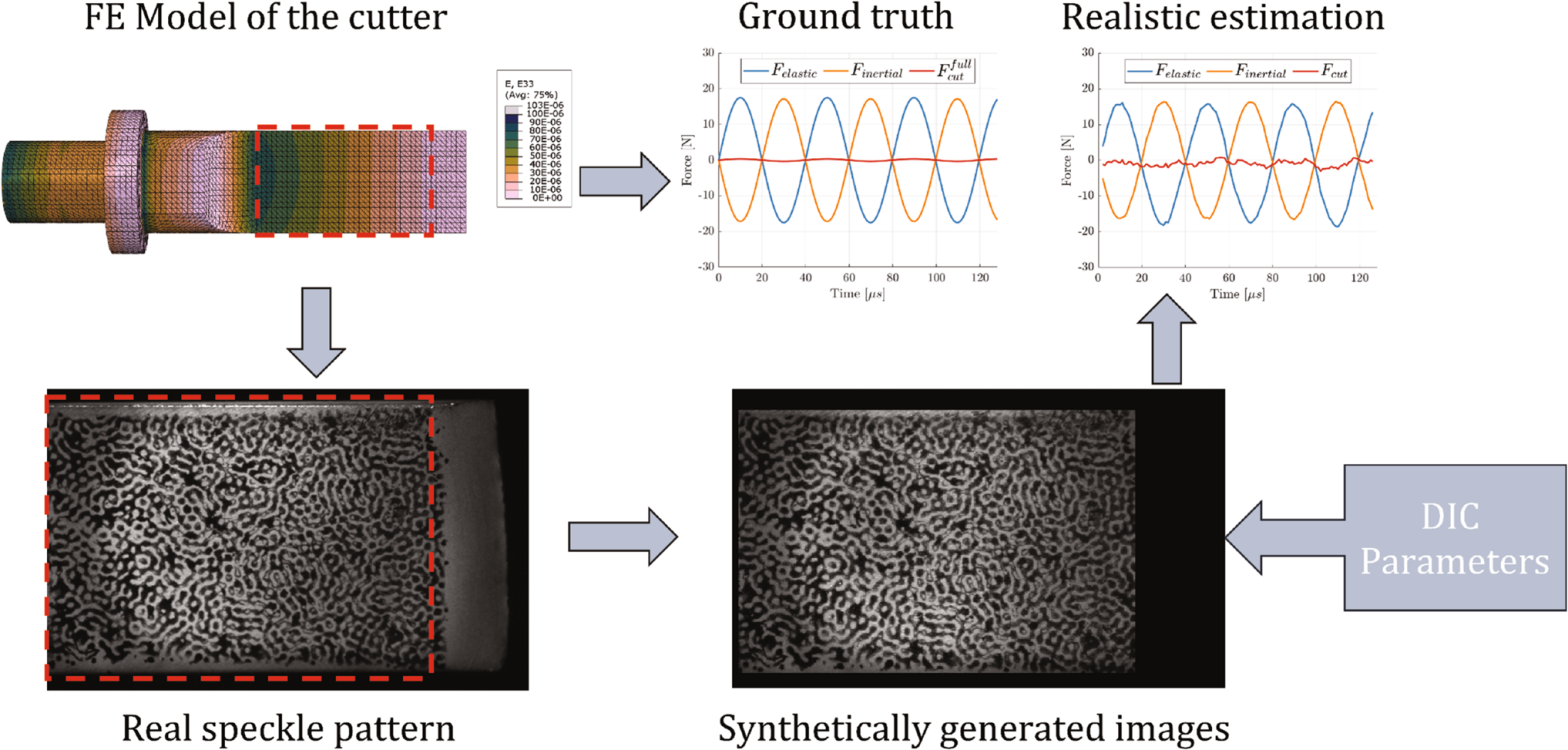
Schematic of the DIC and post-processing parameter optimisation. Kinematics are taken from the finite element (FE) model and used to produce synthetic greyscale images encoding the FE results. These images are processed using the proposed pipeline and result in an estimate of the cutting force, that represents a realistic estimate of the experimental uncertainties. The parameters of the data processing pipeline are optimised to minimise the errors on the calculated force

**Fig. 5:**
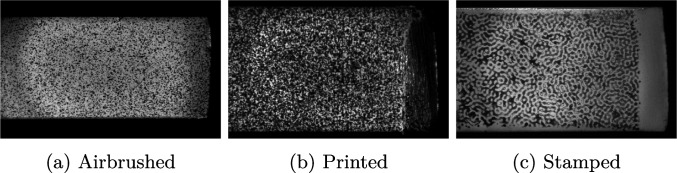
Different types of speckle patterns employed in this study

As discussed previously, digital image correlation results are affected by a number of parameters selected by the user, such as the subset size. These parameters have a profound effect on the final results, therefore there is a need for a rational and objective method of their selection. The method employed in this study was developed by Rossi et al. [26, 27] and relies on the synthetic deformation of images using finite element deformation data (thereafter referred to as ‘Image deformation’) [28]. In short, a finite element (FE) model of the test was built, with known reference parameters (such as boundary conditions and material properties). The displacement fields from this model were used to deform a reference image of the selected speckle pattern, using optical flow rules, and produce synthetic images that encode the FE kinematics. Grey level noise was added to the generated images, to replicate experimental errors accurately. The level of noise was obtained from experimental stationary images. The proposed data processing pipeline was used with these simulated images and the resulting quantities were compared against the known inputs (see Fig. 4). The processing parameters were optimised to produce the best settings for the given experimental setup, leading to the lowest errors. In this study the finite element results were used to deform real speckle images to select the best combination of processing parameters. The parameters that were optimised were: subset size, subset shape function (affine or quadratic) and strain method calculation (local method: strain window and strain polynomial order, global method: polynomial order). For each combination, the cutting force was calculated and the bias (defined as mean error between the measured and expected force) and the variation (defined as standard deviation of the error) were recorded. In total, three speckle patterns were tested: one obtained with an airbrush, one printed directly on the blade using a flatbed printer and one transferred with a soft stamp, see Fig. 5. Ideally, speckles should be 3 px to 7 px in size uniformly distributed between light and dark colours. For each speckle pattern, a hundred combinations of the different parameters were investigated.

### Experimental Setup

The experimental setup consisted of high-speed cameras that recorded the vibrations of an in-house built Langevin-based cutting device operating at 25 kHz. The tool was driven by a commercial broad-band power generator (PDUS210-400, Piezodrive). Two types of cameras were used: for 2D-DIC, a single Shimadzu HPV-X (frame rate up to 5 Mfps) was used recording at 1 Mfps; for stereo-DIC where two cameras are needed, two i-Speed 513 (iX) cameras were used with recording rate set to 75 kfps. The Shimadzu camera was equipped with a high magnification lens (12X Zoom, Navitar), while the iX cameras were fitted with Sigma 105 mm macro lenses. The illumination was achieved by a pulsed laser (Cavilux Smart UHS, Cavitar) synchronised with the camera acquisition. Additionally, the generator’s output was monitored via an AC-coupled port and recorded together with a DC signal coupled to the camera acquisition using a picoscope.

**Fig. 6:**
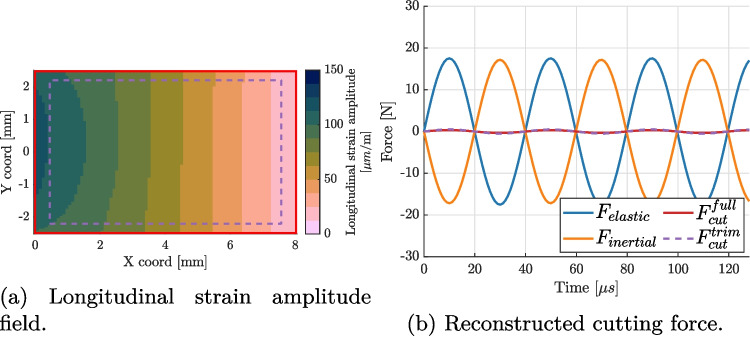
Numerical verification of the proposed method from pure FE data. (**a**) shows the longitudinal strain amplitude fields as extracted from the finite element model. (**b**) shows the measured cutting force, which matches the expected zero force

**Fig. 7:**
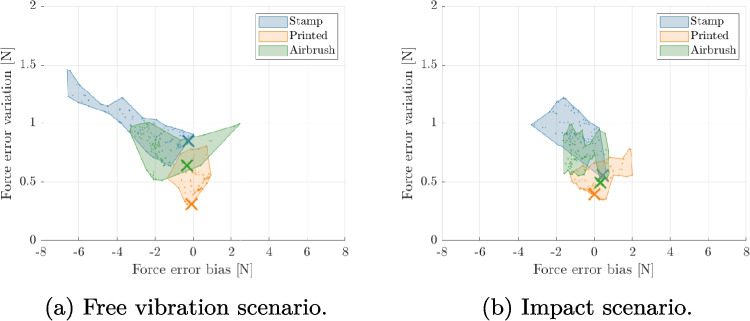
Identified error estimates due to different DIC processing parameters using the image deformation procedure. Small points indicate all considered parameter combinations, the regions indicate the general spread of the results and the large crosses visualize the errors for the optimal set of parameters leading to the smallest combined error

For the validation experiments, free vibration conditions were investigated in which no cutting force is present. In that case, the tool was placed on a set of linear stages to maintain stability of the assembly. For the cutting experiments, the tool was suspended off a motorised cutting platform (designed internally). Its vertical position was controlled by a microcontroller-driven actuator. The static force between the tool and the workpiece was measured by means of a load cell placed between the tool and the gantry, and the signal from the load cell was used to trigger the camera when the static load exceeded a predefined value (*e.g.* 5 N).

## Results and Discussion

### Image Deformation Study

The first step was to numerically verify the proposed method of calculating the cutting force. The finite element displacements, corresponding to the flat part of the blade, were extracted from Abaqus to Matlab for processing. The displacements were used to calculate strains and accelerations and used to calculate the cutting force. We defined two auxiliary forces, elastic and inertial, that are useful for visualising the results:

19 $$\begin{aligned} F_{elastic}= &   \text {IVW}/L \end{aligned}$$

20 $$\begin{aligned} F_{inertial}= &   \text {AVW}/L \end{aligned}$$

with a simple relation between the three forces: $F_{cut} = F_{elastic} + F_{inertial}$. The amplitude of the longitudinal strain field directly output from the FE model (*i.e.*, without image deformation) is shown in Fig. 6(a). The strains and accelerations were used to calculate all three forces ($F_{cut}$, $F_{elastic}$ and $F_{inertial}$) and the results are presented in Fig. 6(b). The figure shows that the method correctly identified the balance between elastic and inertial forces, with zero cutting force, as expected on a free vibrating blade. This verifies the implementation of the calculations of the different auxiliary forces. The small undulation of the cutting force is due to the fact that the finite element model is 3D and does not exactly lead to constant strain and acceleration values through the thickness, but the amplitude is small enough so that it can be safely neglected compared to the target cutting forces (a few Newtons at least). One practical concern was that digital image correlation is not capable of producing data close to edges of ROI. Therefore, in practice, a trimmed ROI has to be used. It was investigated if such trimming (see the dashed outline in Fig. 6(a)) has a significant effect on the results. While the top and bottom edges do not significantly impact the average values measured in the ROI, the region between the ROI and the tapered end of the blade has to be accounted for explicitly. To do that, it is included as the part of the overhang and its volume is added to the overhang volume. Once this is accounted for, the reconstructed force matches the one from the full region of interest.

With the method now verified, the finite element fields were used to generate synthetic images for digital image correlation. Grey level noise with zero mean and standard deviation of 250 (grey levels, 16 bit digitization), was added to the generated images. The magnitude of the noise was based on an estimate from real experiments, when analysing a set of static images. The images were then processed using MatchID 2D using a hundred different combinations of DIC parameters.

**Fig. 8:**
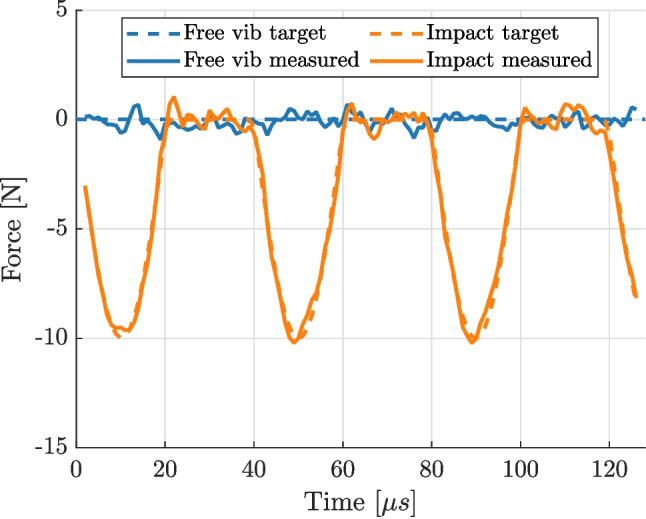
Comparison of the expected and reconstructed forces for two scenarios: free vibration and impact. The application of the image deformation process introduces some noise to the measurement which can be used as a realistic estimate of the experimental uncertainty

The optimisation procedure consistently promoted the use of larger subsets (the best was 33 px), affine shape functions and strain calculation using the ‘global’ method with 2^nd^ order polynomials. The resulting force biases and variations are plotted in Fig. 7. Each combination has been presented as a dot and the one leading to the lowest overall error (defined as root mean square of bias and variation) is marked with a cross. From the figure, it is clear that the speckle pattern has a significant effect on the overall quality of the measurements, so does the combination of DIC parameters. The best achieved reconstruction is presented in Fig. 8. Clearly, the identified forces match very well the reference input. The image deformation procedure introduced some noise but the overall error is very small for both free vibration and impact scenarios.

### Free Vibration with 2D DIC

**Fig. 9:**
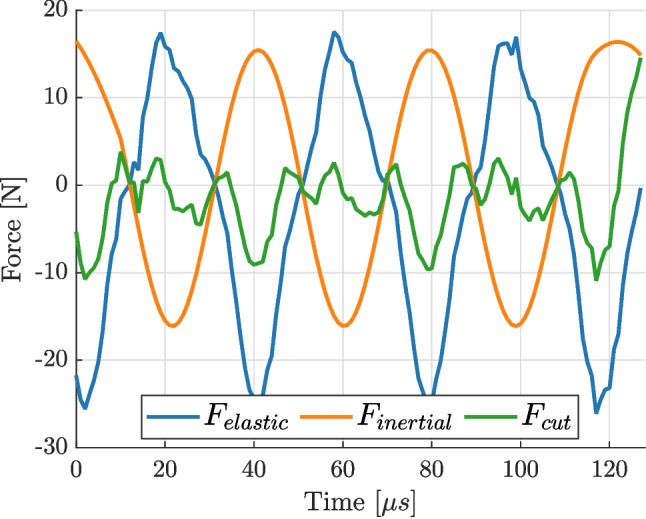
Estimated cutting force under free vibration conditions using 2D DIC

At the first step, free vibration experiments were performed to validate that a zero cutting force could be determined experimentally. The material properties were selected based on the datasheet from the supplier of the Titanium alloy used to build the cutting tool: Young’s modulus of 113.7 GPa, Poisson’s ratio of 0.31 and mass density of 4430 kg m$^{-3}$. The results from one such tests are presented in Fig. 9. The figure confirms that the method is capable of detecting zero cutting force experimentally with slightly higher noise than anticipated from the image deformation study. In this example, there seems to be some non-zero signal when the elastic force is in the negative phase, even though the blade is freely vibrating in air. It was also noted that the results were not consistent between repetitions of the same experiment. Often, the identified force would not be zero; instead it would be harmonic with the frequency and the phase matching that of the elastic and inertial forces. After inspection of many experiments, it was found that the magnitude of the inertial force was consistent between tests, however, the elastic force varied from test to test. Further investigation found that the strain values varied from test to test, not only in amplitude, but also in the mean value versus time.

There were two plausible reasons for such observation: the first one was that there was some small motion of the blade, in the direction of the camera, *e.g.* due to misalignment of the experiment, or due to vibration of the blade. Even 1 µm of out-of-plane displacement would cause spurious strains of approximately 11 µm m$^{-1}$, significant in comparison to the signal that is measured. On the other hand, if the blade was vibrating with an out-of-plane bending mode, the underlying assumption of constant stress through the thickness would be violated. The strains measured by the camera on the surface would not be representative of the membrane strains and therefore, the equilibrium equation would be biased.

These concerns could not be resolved with a single camera and therefore, such configuration was not capable of measuring the cutting force experimentally. To reduce the effect of the out-of-plane motion, a second camera was introduced and stereo DIC was employed to measure the kinematics. In that case, the system would no longer produce errors due to the out-of-plane motion and the bending could be accounted for by estimating the membrane strains using thin plate theory.

### Application of Stereo DIC

#### Free vibration validation

**Fig. 10:**
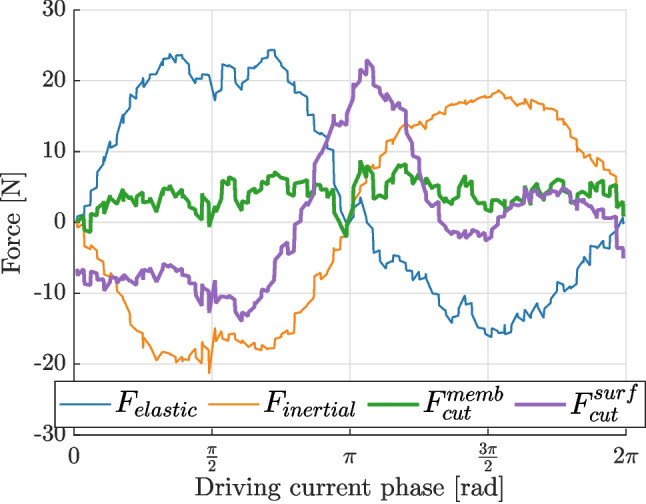
Cutting forces identified with stereo DIC, with and without taking into account bending effects

**Fig. 11:**
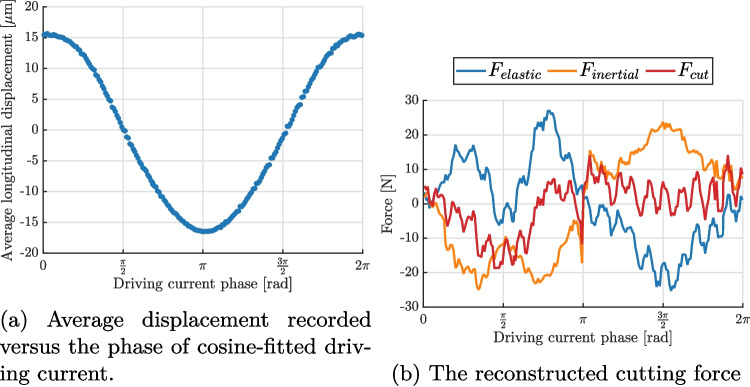
Results of a cutting experiment. (**a**) Time-history of a longitudinal displacement of the blade, (**b**) reconstructed cutting force during the experiment showing non-zero force during vibration

As discussed in the previous section, 2D DIC is sensitive to small out-of-plane motion, therefore a stereo DIC system was used to explicitly quantify this effect. Accelerations were calculated according to the procedure outlined in Section “Time registration of stereo images”, while out-of-plane displacements were used to account for the blade bending. The measured deflections were fitted with a 2D quadratic surface, which was differentiated twice in space to yield average curvatures across the entire ROI. The curvatures and the measured surface strains were then combined to calculate the membrane strains, according to equation (7).

The effect of the bending strains on the measured cutting force is seen clearly in Fig. 10: when the cutting force is calculated using surface strains (purple line), it has a periodic form. On the other hand, when the strains are calculated at the mid-plane, the reconstructed cutting force is correctly identified as zero. This observation was consistent across multiple experiments, therefore the proposed methodology was validated experimentally using stereo measurements.

#### Ultrasonic cutting

The method was validated for the case when no cutting force is present, which was possible due to the fact that the vibrations are steady-state and recording at slower rates can still produce accelerations. This assumption may not hold under cutting conditions, as it is likely that a significant non-linearity may be present when the blade disengages from the material and then impacts it in the following cycle. Nevertheless, the cutting force was investigated as for free vibrations, noting that the reconstructed accelerations might be biased. The non-linearity should manifest itself in the measured signal. While for the free vibration conditions, the average displacement and strain has a clear sinusoidal shape, it was thought that this might not be true for the cutting scenario where local temporal variations are superimposed onto the carrier frequency.

An experiment was performed using the method outlined in Section “Experimental Setup”. A 25 kHz tool was driven into a piece of bovine cortical bone, excised from the tibia, at the rate of 50 µm s$^{-1}$ and the recording was triggered when the load cell detected 5 N of static loading. The tool was driven with the Piezodrive generator, programmed to maintain a constant current amplitude of 0.6 A, leading to a vibration amplitude on the order of 30 µm peak-to-peak. The blade was observed using two iX cameras, recording at 75 kfps with a window of $222 \times 392$ px$^{2}$. 200 images were collected over 3 ms, which corresponds to approximately 82 cycles of vibration. Due to a significant rigid body motion between the static images (taken before the experiment) and the cutting experiment, the images were correlated against an image corresponding to peak velocity, *i.e.* minimum displacement. As a result, the measured strains (and forces) are expressed as values relative to that image, rather than absolutes.

**Fig. 12:**
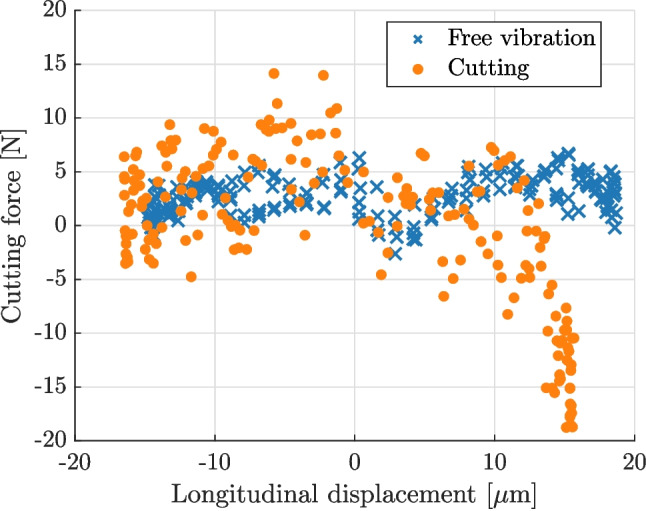
Comparison of force versus extension data for free vibration and cutting experiments. Clear compression force is detected when the blade extends into the workpiece

The measured average longitudinal displacement as a function of the driving current phase is shown in Fig. 11(a). The figure shows a clear sinusoidal shape of the measured displacement, with a slight asymmetry between the positive and negative phases. This result justifies the use of the same data processing as for the free vibrations. The cutting force and related non-linearities only marginally impact the global sine response of the tool. The kinematic data were then processed to obtain acceleration and strain fields at each image, which were then used to calculate the cutting force, represented in Fig. 11(b). The figure shows that the cutting force is detected and correlated with the vibration cycle: it peaks at the driving current phase of $\pi /2$, which corresponds to the maximum extension of the tool. The maximum cutting force reached is approximately 15 N. An interesting comparison can be made against the free vibration condition when the reconstructed cutting force is plotted against the average longitudinal displacement, as shown in Fig. 12. In the case of free vibration, the trend is close to a constant line, close to 0 N independently of the displacement as expected. On the other hand, during the cutting experiment the force is close to zero for the majority of the cycle. However, as the blade reaches maximum extension, the measured force drastically increases, which is consistent with the hypothesis of vibro-impact. It is worth noting that the measured peak force is significantly higher than the one measured with a load cell connected to the cutter (about 5 N), as it is too far from the cutting site to record accurate cutting forces in the inertia-driven scenario.

The main limitation of this method comes from the imaging practicalities. To achieve better results, cameras with higher frame rates and spatial resolutions are needed. Another important aspect is the required magnification: as the cutting tools are small, the optics have to be sufficiently strong which limits the applicability of stereo setups. If cameras with more pixels were available, then to achieve better spatial resolution smaller speckle pattern would need to be deposited, which is another challenging problem.

## Conclusions and Future Work

In this contribution we presented an image-based method for measuring the vibro-impact force in the context of ultrasonic cutting of bone which relies on observing the cutting device with a high-speed camera. While in this study we focused on the longitudinal force, another set of virtual fields can be selected to measure the transverse force at the same time, therefore yielding a more complete information about the interaction between the tip and the material. The advantage of this method is that it is contactless, *i.e.* it does not influence the dynamics of the system and that it enables time-resolved measurements directly at the cutting site.

The main conclusions from this study are:

1. Cutting force can be successfully measured from high-speed images for a chiselling type device by utilizing digital image correlation and the virtual fields method. The method was validated numerically and experimentally in a free vibration scenario (known zero cutting force), before applying it to cutting of bovine bone.
2. The measured force is dynamic (changes with time) and the peak value is much higher than the output of the load cell in the experiment. Peak cutting force was measured at approximately 20 N at the extension of 16 µm.
3. A detailed study of factors affecting image-based measurement was performed and it was found that DIC parameters have significant impact on the measured force and therefore they have to be optimised using synthetic image deformation procedure. Likewise, the DIC pattern is an important factor for the overall quality of the measurements.
4. Stereo DIC had to be utilized to obtain reliable results. The initial assumption of 2D mechanical fields was proven to be insufficient and bending strains had to be accounted for by measuring the curvature of the blade during the experiment.
5. Tracking current from the ultrasonic generator is an efficient method of registering images when imaging at frequencies insufficient for direct measurement of accelerations through temporal derivative of displacements.

The developed methodology opens new avenues of exploring the mechanical interaction between ultrasonic cutting devices and the processed materials and could be used in the future to investigate the effect of process parameters on the developed force. The time-resolved force is an interesting quantity to cross-reference against other important aspects of bone cutting, such as the chip morphology, the cut surface quality or the biological responses after the cut was performed. Using this method it would be possible to study the effect of feed rate, or vibration amplitude on the measured peak force and better understand the cutting mechanism associated with ultrasonic tools. The method is general and not limited to a particular configuration, therefore an interesting study would be to apply it to another cutting device, *e.g.* one designed to perform sawing motion.

## Data Availability

Dataset available on request.
